# Sulfur-mediated bacteria outperform glycogen-accumulating organisms in carbon-deficient wastewater: Key role of influent C/S^0^ ratios

**DOI:** 10.1016/j.fmre.2023.10.024

**Published:** 2024-01-14

**Authors:** Boyi Cheng, Lei Chen, Lichang Zhou, Qingshan Lin, Jinqi Jiang, Hui Lu, Lei Miao, Xiaonan Feng, Zongping Wang, Guanghao Chen, Gang Guo

**Affiliations:** a School of Environmental Science and Engineering, Huazhong University of Science and Technology (HUST), Wuhan 430074, China; b School of Environmental Science and Engineering, Sun Yat-sen University, Guangzhou 510006, China; c Department of Civil & Environmental Engineering, The Hong Kong University of Science and Technology, Hong Kong 999077, China

**Keywords:** Elemental sulfur, Competition, Sulfur-mediated bacteria, Glycogen-accumulating microorganisms, Carbon-deficient wastewater

## Abstract

Biological nutrient (including nitrogen and phosphorus) removal is often hindered by the lack of organic carbon (C) sources, which can result in the excessive growth of glycogen-accumulating organisms (GAOs), even in sulfate-containing wastewater. Elemental sulfur (S^0^) has been considered as an economical and energy-efficient electron donor to achieve biological nutrient removal from wastewater. In this study, the long-term effects of C/S^0^ ratios on the competition between sulfur-mediated bacteria (SMB) and GAOs was investigated to explore the feasibility of using S^0^ to partially replace carbon and suppress the proliferation of GAOs in biological treatment systems. Four parallel bioreactors were continuously operated for approximately 100 days and fed a substrate consisting of 400, 200, 100, and 0 mg acetate-COD/L, in addition to 0.53 g S^0^/cycle (equal to 400 mg COD/L); the acetate-COD concentrations corresponded to C/S^0^ ratios of 0.28 (R1), 0.14 (R2), 0.07 (R3), and 0 (R4), respectively. The results showed that a relatively high C/S^0^ ratio of 0.28 (in R1) and low C/S^0^ ratios of 0.07 (in R3) and 0 (in R4) enhanced 1) S metabolism, especially anaerobic S^0^ reduction to form S^2−^, which was mediated by sulfate/sulfur-reducing bacteria (SRB/S^0^RB, e.g., *Desulfobacter and Desulfuromonas*), or 2) S^0^ oxidation to form sulfate, which was mediated by sulfur-oxidizing bacteria (SOB, e.g*., Thiobacillus*) to promote their growth over GAOs (e.g., *Candidatus_Competibacter*). However, when the C/S^0^ ratio was 0.14 in R2, this reactor displayed a GAO phenotype rather than a SMB phenotype since GAO communities have a higher acetate uptake rate than SMB. Thus, an appropriate amount of S^0^ addition supported the competitiveness of SMB over GAOs. Finally, the potential mechanism about competition between the two communities (SMB and GAOs) was revealed. This study provides a new mechanistic strategy to promote the proliferation of SMB over GAOs in carbon-deficient wastewater.

## Highlights

• Continuous operation of four SBRs receiving various C/S0 ratios.
• Favoring of SMB's outperformance of GAOs by appropriate C/S0 ratios.
• S° could replace partial carbon and avoid overgrowth of GAOs in nutrient removal.
• Reveal of potential mechanism about competition between SMB and GAOs.
• Higher acetate uptake rate for GAOs compared with SMB.

## Graphical abstract

**Figure:**
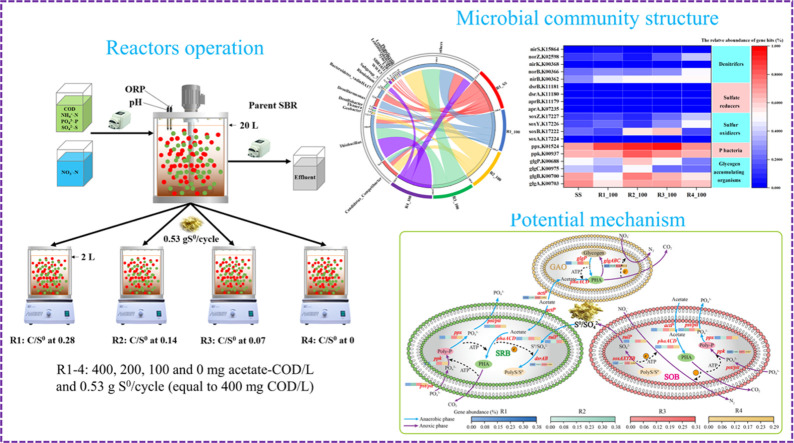

## 1 Introduction

Urban wastewater treatment is generally challenged by the insufficient availability of organic carbon sources (e.g., < 200 mg COD/L), especially in southern China, which can significantly affect biological nutrient (nitrogen [N] and phosphorus [P]) removal [1,2]. A preliminary solution to this problem was the addition of organic matter (e.g., methanol), but the costs and carbon emissions from methanol use did not make this strategy sustainable [3]. In recent years, a series of sulfur (S) species, including SO_3_^2−^, S_2_O_3_^2−^, S^0^, and S^2−^, were used by sulfur bacteria as electron donors/co-carriers, to replace organic carbon, for N and P removals in urban wastewater treatment. For instance, several researchers have reported achieving sulfur oxidation-autotrophic denitrification coupled with heterotrophic denitrification for N removal in insufficient carbon wastewater treatment [4], [5], [6]. Recently, several studies have reported the use of sulfur sources to enhance biological P removal from wastewater [7,8]; these include the development of denitrifying sulfur-conversion-associated enhanced biological P removal (DS-EBPR) systems [9]. In this novel process, sulfur-reducing bacteria (SRB) and sulfur-oxidizing bacteria (SOB) simultaneously remove C, N, and P pollutants with limited sludge production. The autotrophic or mixotrophic metabolism properties of SRB and SOB present possibilities for nutrient removal during wastewater treatment under low-carbon conditions.

However, unfavourable environmental or operating conditions can lead to the excessive growth of glycogen-accumulating organisms (GAOs), e.g., *Competibacter* and *Alphaproteobacteria* GAOs, resulting in unstable biological P removal performance [10]. Control strategies are required to suppress the proliferation of GAOs and achieve stable/optimal DS-EBPR performance, but such strategies require an understanding of how operating or environmental conditions affect the competition between functional bacteria (i.e., SRB and SOB) and hostile GAOs. In conventional EBPR systems, certain operating and environmental conditions, including pH, type of carbon source or temperature, have been identified as the key factors influencing the competition between phosphate-accumulating organisms (PAOs) and GAOs [11]. Although the short-term effects of the above factors have been examined in the DS-EBPR process [12,13], they have been shown to fluctuate in previous studies [8,9]. Thus, it is necessary to more comprehensively examine the optimal conditions for the growth of SMB over GAOs to metabolize S, C, N, and P under low-carbon conditions, which has not been explored to date.

As the zero-valent form of sulfur and the species with intermediate valence in the geochemical sulfur cycle, elemental sulfur (S^0^) is inexpensive and earth-abundant. It has widely been used for organic matter removal, sludge reduction, and heavy metal precipitation via S^0^-driven autotrophic denitrification processes (S^0^AD) or S^0^ reduction processes (S^0^R) [4,5,14]. In particular, S^0^ has been reported to be a suitable substrate, particularly for SRB and SOB growth, but seldom for GAO growth [10]. Therefore, the aim of this study is to investigate the competition between SMB (including SRB and SOB) and GAOs under long-term operation by using S^0^ to partially replace carbon as the electron donor. The effects of different influent mass ratios of carbon and S^0^ (C/S^0^) of 0.28 mg C/mg S^0^ (in R1), 0.14 mg C/mg S^0^ (in R2), 0.07 mg C/mg S^0^ (in R3), and 0 mg C/mg S^0^ (in R4) on the competition between SMB and GAOs were studied in a failed DS-EBPR system. During the experiments, the reactor performance, including the C, N, and P removal efficiencies and S conversion rate, production of internal polymers (e.g., glycogen, poly-β-hydroxyalkanoates [PHA], polysulfides (polyS]), key sludge characteristics, and microbial community dynamics, were evaluated. Finally, strategies based on manipulating the S^0^ addition for the process optimization were proposed. The outcomes of this study elucidate the competition dynamics of SMB and GAOs and present strategies to suppress the proliferation of GAOs to optimize system performance, especially under low-carbon conditions.

## 2 Materials and methods

### 2.1 Operation of the parent sequencing batch reactor (SBR)

A 23.5 L laboratory-scale parent sequencing batch reactor (SBR) with a 20 L working volume (75 cm height and 20 cm diameter) was operated under alternating anaerobic and anoxic conditions for approximately 580 days to cultivate different microbes (SRB, SOB and GAOs) based on a previous work [15]. The temperature of the reactor was set to 30 ± 1 °C, and the pH was kept between 7.0∼7.9. The mixed liquor suspended solids (MLSS) levels were kept between 5∼8 g/L, and the sludge retention time (SRT) was set to be approximately 30 d by periodic sludge discharge. During the later stage of operation, the average anaerobic sulfate reduction and anoxic sulfate production in the parent SBR were 42.5 mg S/L and 37.2 mg S/L, respectively; the rates of anaerobic COD consumption and anoxic nitrate consumption were 27.4 mg COD/(L·h) and 17.4 mg N/(L·h), respectively, and the final P removal was approximately 1.5 mg P/L. Furthermore, the microbial community in the reactor changed from merely SRB (*Desulfobacter*, 3.25%) and SOB (*Thiobacillus*, 12.5%) to SRB (*Desulfobacter*, 3.1%), SOB (*Thiobacillus*, 13.5%), and GAOs (*Candidatus_Competibacter*, 13.2%), indicating that SMB and GAOs coexisted in the system in relatively equal abundances.

### 2.2 Long-term effects of different C/S0 ratios on the competition between SMB and GAOs

The long-term effects of the C/S^0^ ratios on the competition between SMB and GAOs were investigated using four SBRs, each having a working volume of 2 L (see Fig. S1 in the Supporting Information (SI)). A mixture of 8 L of sludge and liquid was withdrawn from the parent SBR at the end of the anoxic phase on Day 580 and allowed to settle for 1 h to discharge 4 L of supernatant. Then, the residual sludge was washed twice with deionized water, divided evenly and transferred into four reactors. Subsequently, 1 L of synthetic wastewater was rapidly added to each reactor at the beginning of the cycle, which resulted in a mixed liquor volatile suspended solid (MLVSS) level ranging between approximately 3.9∼4.4 g/L in all four reactors. The SRT was set to approximately 30 d by periodic sludge discharge from each reactor. These four reactors were independently operated in a cyclical anaerobic-anoxic pattern. Each operating cycle consisted of i) a feeding phase that consisted of adding 1 L of synthetic wastewater for 10 min, ii) an anaerobic phase that varied in duration in different reactors (see Table 1), iii) a phase in which a sodium nitrate solution of 2 g N/L was introduced to achieve a total nitrate concentration between 25 and 40 mg nitrate-N/L, iv) an anoxic phase that varied in duration in different reactors (see Table 1), v) a settling phase of 30 min, vi) a phase in which 1 L of the supernatant was decanted for 10 min, and vii) an idle phase of 30 min. The durations of the anaerobic and anoxic phases were varied to ensure that complete S, C, N, and P bioconversions were achieved [10].

**Table 1:** **Experimental setup and operating conditions in R1–R4**.

Reactor	Phase	Days	Cycle length (h)	Anaerobic phase			Anoxic phase	
				Time (h)	HAc (mg COD/L)	S^0^ dose (g)	Time (h)	NO_3_^−^ dose (mg N/L)
R1	Phase I	0∼80	10→15	8→9	400	0.53	2→6	40→45
	Phase II	81∼100	15	9			6	45 ± 5
R2	Phase I	0∼80	10→6	8→3	200	0.53	2	30→45
	Phase II	81∼100	6	3			2	45 ± 5
R3	Phase I	0∼80	10→7	8→3	100	0.53	2→3	25→45
	Phase II	81∼100	7	3			3	45 ± 5
R4	Phase I	0∼80	10→49	8→1	0	0.53	2→48	25→45
	Phase II	81∼100	49	1			48	45 ± 5

The arrows represent the changes of the experimental parameters and operating conditions.

Based on a previous study [10], the synthetic wastewater for the four reactors was prepared from 20 mg of NH_4_^+^-N/L, 20 mg of PO_4_^3−^-P/L, 180 mg S/L of sulfate, and different concentrations of acetate. The COD concentration varied among the different reactors: 400 mg COD/L in R1, 200 mg COD/L in R2, 100 mg COD/L in R3, and 0 mg COD/L in R4. At the beginning of each cycle, 0.53 g of S^0^ (corresponding to approximately 400 mg/L of COD) was added to the four reactors, and to simulate the carbon-deficient wastewater commonly found in practice, the organic carbon concentration in R2-R4 was therefore decreased to 200, 100 and 0 mg S/L, respectively, so the corresponding mass ratios of C/S^0^ were 0.28, 0.14, 0.07 and 0 in R1-R4. The influent pH was adjusted to approximately 7.5.

The four reactors were continuously operated for more than 100 days and divided into two stages, that is, the adaptation stage from Day 1 to Day 80 (phase I) and the stable stage from Day 81 to Day 100 (phase II) (see Table 1). During the long-term operation, four to six mixed samples of 2–4 mL each were periodically taken from each reactor during the anaerobic and anoxic phases of each cycle to monitor the reactor performance. These samples were first filtered through 0.22-µm pore-size filters for analysing acetate, ortho-P (PO_4_^3−^-P), sulfate, sulfide, thiosulfate, nitrate, and nitrite. The cyclic behaviour of each reactor was examined in cyclic tests at approximately 20-day intervals. In each test, 6–10 samples of 20 mL each were collected from every reactor and filtered through 0.45-µm pore-size filters, and the filtrate was used to measure key anions in the liquid; the residual solids were used to analyse PHAs, glycogen, and polyS. Furthermore, 20 mL samples collected at the end of the anoxic phase were analysed for 1) sludge characteristics by several methods, including scanning electron microscopy (SEM), X-ray diffraction (XRD), and X-ray photoelectron spectroscopy (XPS); and 2) microbial community by using Illumina high throughput sequencing of the 16S rRNA genes.

### 2.3 Analytical methods

The key anions in the bulk liquid, including acetate, nitrite, nitrate, phosphorus, sulfate, and thiosulfate, were determined by ion chromatography (DX-120, USA). The MLSS, MLVSS and sulfide were measured according to standard methods [16]. The pH and ORP were measured using a digital pH meter (pHS-25, China) and an ORP meter (ORP-501, China), respectively. The PHA was measured by gas chromatography‒mass spectrometry (Agilent 7890A-5975C, USA) according to Oehmen's method [17]. Glycogen was analysed by the anthrone method according to Jenkins et al. [18]. The polyS was measured by using the sulfite method [19]. The morphology and microstructure of each reactor's sludge on Day 100 were examined using SEM (JSM-IT200, Japan). The chemical composition of the precipitates in the sludge sampled on Day 100 was determined by X-ray diffraction (X'pert Pro MPD, Netherlands) and X-ray photoelectron spectroscopy (AXISULTRA DLD-600 W, Shimadzu, Japan) (see more details in Table S1 in SI).

### 2.4 Microbial community analysis and gene prediction

To monitor the shift in the microbial community, sludge samples were taken on Days 0, 10, 50, 80 and 100 from each reactor and subsequently sent to Shanghai Majorbio Biomedical Technology Co., Ltd. for 16S rRNA gene analysis. The key processes include DNA extraction, polymerase chain reaction (PCR) amplification, pyrosequencing and data analysis. The functional genetic characteristics of the bacterial communities were predicted via the phylogenetic investigation of communities by reconstruction of the unobserved states (PICRUSt2). The metabolic pathway analysis was conducted based on the Kyoto Encyclopedia of Genes and Genomes (KEGG) database to investigate the biochemical metabolism of the seed sludge and R1–4 according to a previous study [20], and the gene functions were annotated based on the KEGG database using BLASTP and KOBAS 2.0.

## 3 Results and discussion

Under the different influent C/S^0^ mass ratios (mg C/mg S^0^) of 0.28, 0.14, 0.07, and 0 in R1, R2, R3, and R4, respectively, the long-term competition between SMB and GAOs for C, N, P, and S substrates, the typical cycle performance of each reactor, and the microbial community structures were studied.

### 3.1 Effects of C/S^0^ ratios on S-mediated C, N, and P removal performances

#### 3.1.1 Comparison between S-mediated C, N, and P removal performances

**Fig. 1:**
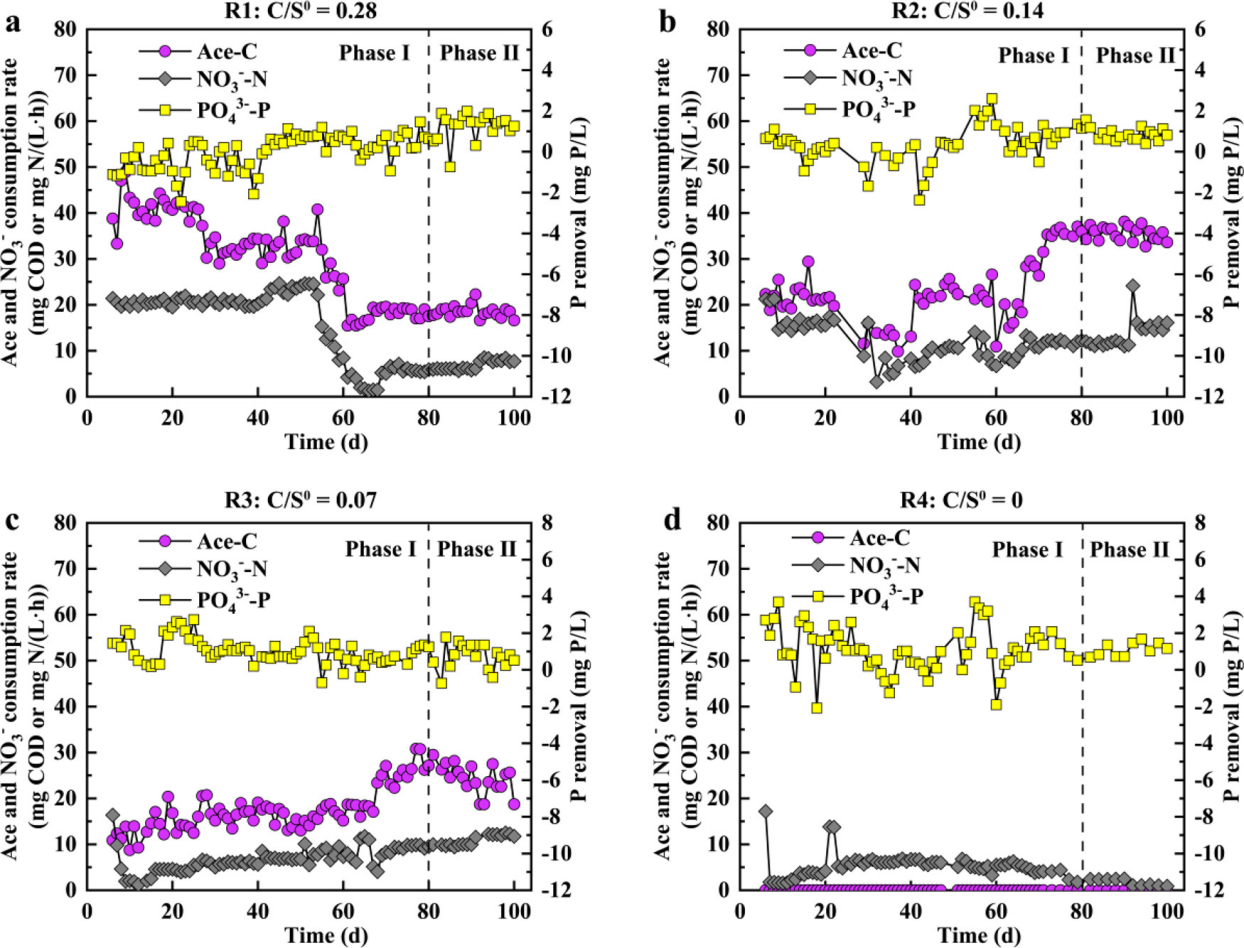
**Changes in C, N and P metabolism in R1–R4 during the long-term operation**.

With respect to C metabolism, acetate was nearly fully consumed in R1–R4 during the anaerobic phase, indicating that the systems could remove C efficiently. The acetate consumption rate significantly changed with the different influent C/S^0^ ratios (see Fig. 1). In R1, the acetate consumption rate gradually decreased from the initial 38.8 mg COD/(L·h) to approximately 17.5 mg COD/(L·h) during phase I on Day 60 and then remained stable at 17.5 mg COD/(L·h) during phase II. In R2, the acetate consumption rate fluctuated in the range of 9–27 mg COD/(L·h) between Day 1–Day 60, and then it increased to approximately 35.5 mg COD/(L·h) thereafter during phase I; it finally stabilized at 36.0 mg COD/(L·h) during phase II. Meanwhile, in R3, the acetate consumption rate increased from 10.8 mg COD/(L·h) to 27.1 mg COD/(L·h), especially from Day 70 to Day 80 during phase I, and then it marginally decreased to 24.3 during phase II. In R4, given that no carbon was added, there was no acetate consumption. Previous studies have revealed that SMB generally have a lower carbon uptake rate than GAOs, particularly when GAOs proliferate in a hybrid system [13]. The significant decrease observed in the acetate consumption rate in R1 suggested that the growth of SMB gradually exceeded that of GAOs. In contrast, the increasing trend in the acetate consumption rate in R2 and R3 suggested that there was relatively high GAO activity.

**Fig. 2:**
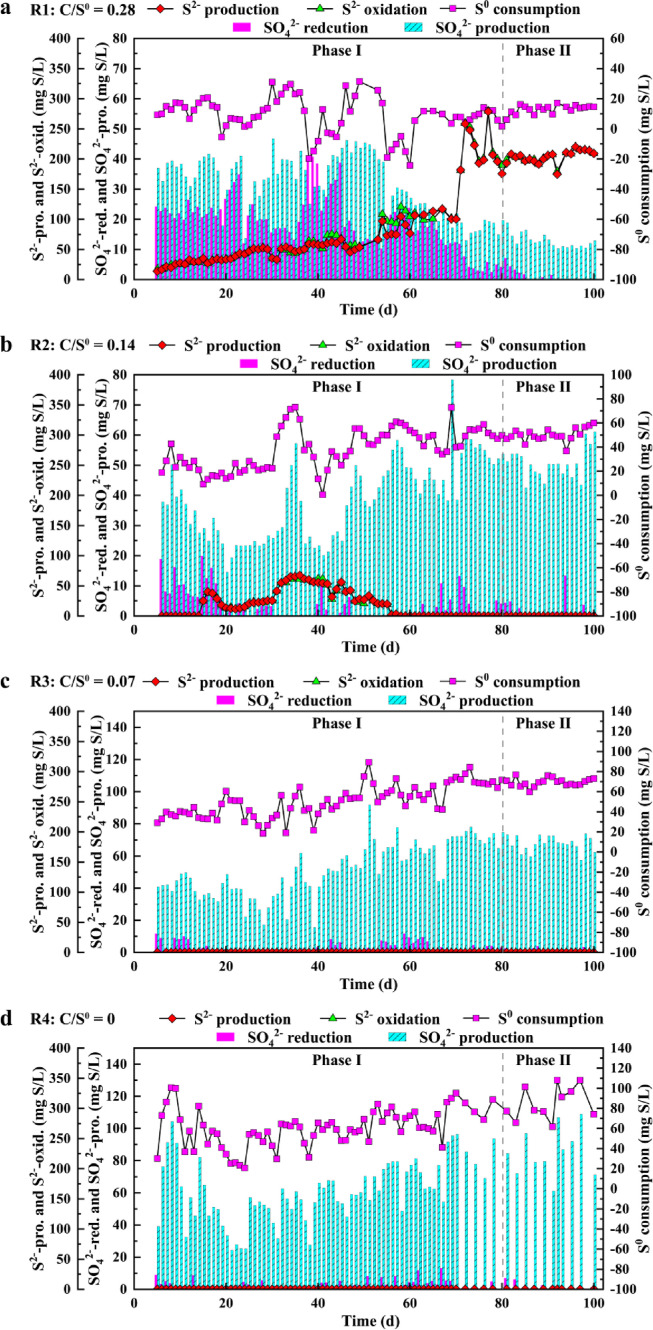
**Changes of S metabolism in R1–R4 during the long-term operation**.

With respect to N metabolism, nitrate was nearly used up during the anoxic phase in R1–R4, showing complete denitrification (i.e., ∼100% NO_3_^−^-N removal). The nitrate consumption rate significantly changed during the long-term operation (see Fig. 1). In R1, the nitrate consumption rate remained stable at approximately 21.4 mg N/(L·h) from Day 1 to Day 55, but it rapidly decreased to 5.4 mg N/(L·h) on Day 80 during phase I, which can be attributed to the high sulfide level of 203.9 mg S/L (Fig. 2), inhibiting the activities of the denitrifiers [21,22]; from Day 80 onwards, it stabilized at 6.8 mg N/(L·h) during phase II. In R2 and R3, the nitrate consumption rate primarily decreased and then gradually increased to between 9 and 16.5 mg N/(L·h). For R4, the nitrate consumption rate remained at relatively low levels of 0.8–6.6 mg N/(L·h), since only S^0^ was added to the influent and S^0^ has low solubility; as a result, mass transfer kinetics was slow [5]. The four reactors, i.e., R1–R4, displayed different nitrate consumption rates because the functional bacteria (Fig. 5) were significantly different after the long-term operation under different influent C/S^0^ ratios and heterotrophic denitrification rates were generally higher than the autotrophic denitrification rate [23].

In terms of P metabolism, the net P removal fluctuated marginally during the long-term operation of the four reactors. When the reactor performances became stable in phase Ⅱ, the net P removal contents were 0.23, 0.47, 0.95, and 1.07 mg P/L, corresponding to P removal efficiencies lower than 10% in R1–R4. Because the seed sludge for the long-term experiments in R1–R4 was enriched with GAOs (*Candidatus Competibacter*, 13.2%) (Fig. 5), which can only use glycogen and PHA as energy and electron carriers, respectively, and cannot carry out poly-P degradation/generation or contribute to P removal, the P removal efficiencies were consistently lower than those of typical DS-EBPR systems. In addition, marginal increases in the net P removals of R1 and R2, with relatively high influent C/S^0^ ratios, were observed during the long-term operation. These increases can be attributed to the fact that the addition of S^0^ restored the activity of the SOB to a certain extent and that some genera of the SOB (e.g*., Thiobacillus*) had sulfur oxidation, nitrate reduction, and P removal functions [24,25].

#### 3.1.2 Comparisons of S conversions

During the long-term operation, the effects of the different influent C/S^0^ ratios on S metabolism, particularly on the conversion of sulfate, sulfide and S^0^ in each cycle, were monitored and analysed, as shown in Fig. 2.

**Fig. 3:**
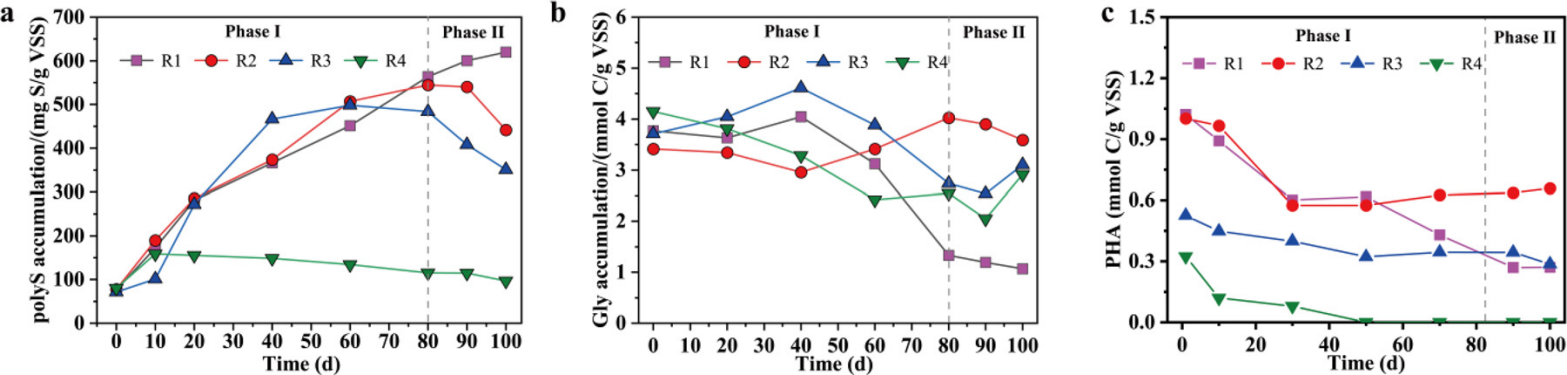
**Changes of poly S (a), glycogen (b) and PHA (c) in R1–R4 during the long-term operation**.

Under anaerobic conditions, the sulfate reduction in R1 ranged between 12.0–40.7 mg S/L between Day 1–Day 40 and then decreased to 4.2 mg S/L on Day 80 during phase I. The sulfate reductions in R2–R4 rapidly decreased from the initial 18.9, 11.7, and 8.9 mg S/L to nearly 0 mg S/L during phase I. Thereafter, the sulfate reduction was nearly nonexistent in the four reactors during phase II. However, the sulfide production in R1–R4 showed a different trend from that of sulfate reduction. The sulfide production in R1 gradually increased from 13.9 on Day 5 to 100 mg S/L on Day 70, but it quickly increased to 175.6 mg S/L on Day 80 during phase I; then, it became stable at approximately 203.9 mg S/L on average during phase II. The sulfide production in R2 primarily increased from naught on Day 5 to 56.2 mg S/L on Day 45, and then it gradually decreased to naught again on Day 58 during phase I, after which it ceased. In comparison, no sulfide production was observed in R3 and R4, which correlated well with the reaction of sulfate reduction. Generally, sulfate is reduced by SRB to form sulfide or polyS [9]. The discrepancy between the observed amounts of sulfate reduction and sulfide production, especially in R1, indicated that sulfide production resulted from S^0^ reduction rather than sulfate reduction. In comparison, in R3 and R4, in which relatively insufficient carbon was present, the activity of SRB or elemental sulfur-reducing bacteria (S^0^RB) was inhibited, and the production of sulfide gradually ceased, producing residual S° for the subsequent reaction stage (i.e., anoxic stage). This observation was further supported by the XRD and XPS analyses of the sludge samples (Fig. 2, Fig. 3, Fig. 4 in SI). Although sulfate is more bioavailable than S^0^, S^0^RB uses S^0^, instead of sulfate, as the electron acceptor for S^0^ reduction to form polyS or S^2−^ [4]. In addition, it was found that in the presence of an insufficient carbon source (e.g., as in R2–R4), S^0^ appeared to be used by SRB/S^0^RB less easily; as a result, they were unable to compete with GAOs.

**Fig. 4:**
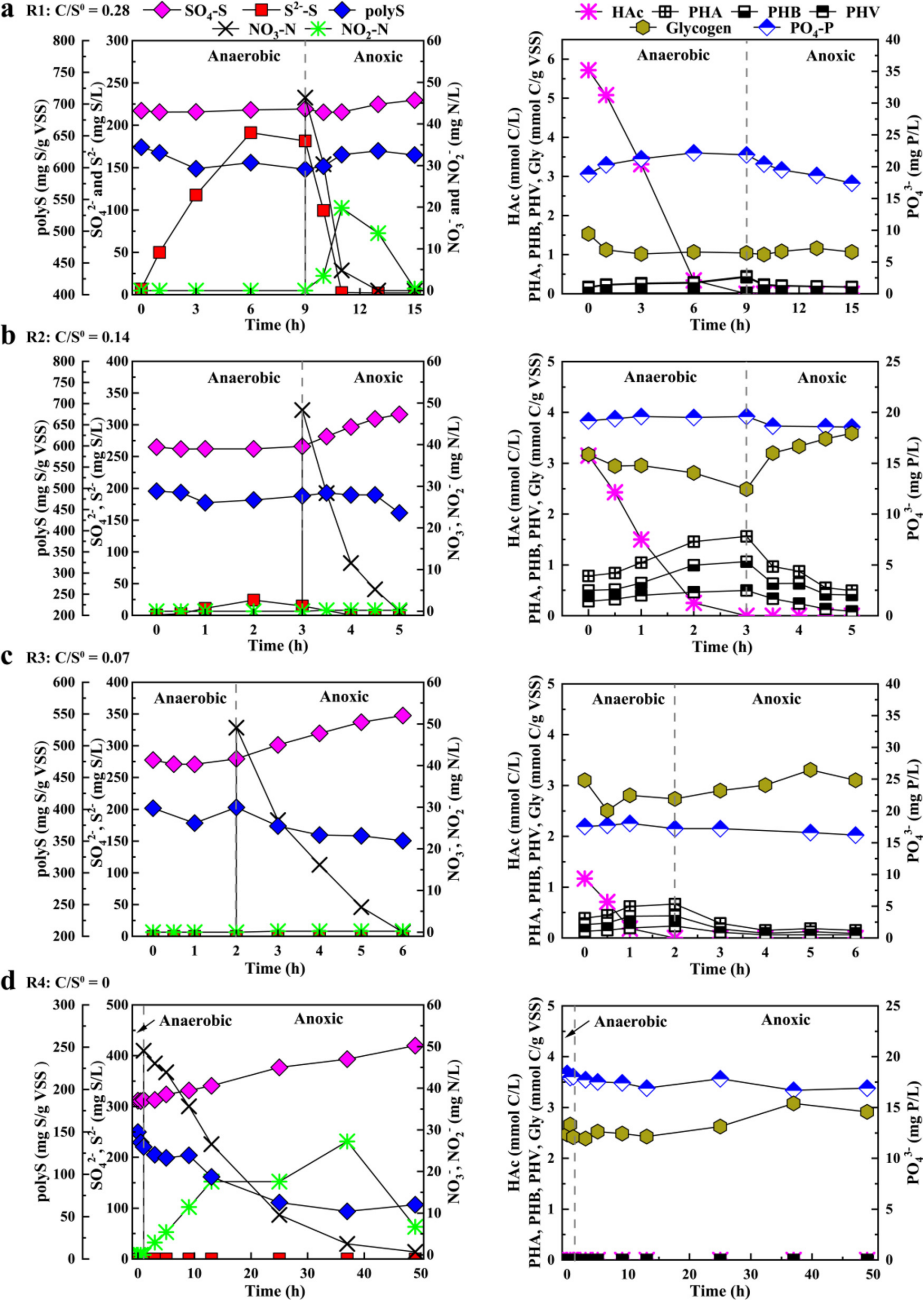
**Cyclic changes of the key compounds in R1–R4 measured in the cyclic tests**.

Under anoxic conditions, SMB (especially SOB) can oxidize polyS/S^0^ or S^2−^ by using nitrate as the electron acceptor for autotrophic denitrification, P uptake and sulfate production. Sulfate production from polyS/S^0^ or S^2−^ oxidation in R1 was mostly between 30 and 45 mg S/L from Day 1 to Day 45, then it gradually decreased to 19.6 mg S/L on Day 80 during phase I. In comparison, the sulfate productions in R2, R3 and R4 marginally decreased and then gradually increased to 53.8, 74.7, and 84.3 mg S/L, respectively, on Day 80. During phase II, the sulfate production in R1–R4 reached relatively stable values of 12.8, 50.5, 67.7, and 85.3 mg S/L on average. Clearly, R1 had less sulfate production than R2–R4 because most of the nitrate was consumed by the SOB for S^2−^ oxidization to polyS/S^0^, rather than to sulfate (see Section 3.2.1). Meanwhile, the sulfate production in R2–R4 continued to increase and reached a relatively high value during phase Ⅱ; this might have occurred because after the reduction of influent carbon sources, more nitrate was used to oxidize S^0^ under the condition of equal nitrate consumption [14], which was consistent with the observation that the amount of sulfate produced was inversely proportional to the influent C/S^0^ ratio. Generally, higher S^2−^ or polyS/S^0^ oxidization can support better growth of SOB [26]. While the sulfate yields were different in R1–R4, the sulfur oxidation reactions mediated by the SOB were not significantly affected given that a large amount of S^0^ was added in each cycle, which favoured SOB over GAOs.

#### 3.1.3 Comparison of internal polymers

Storage polymers, including polyS, PHAs (e.g., PHB and PHV), and glycogen, are the key functionally relevant polymers involved in this mixotrophic system that includes SMB and GAOs. The storage polymers were periodically measured in R1–R4 during the long-term operation (see Fig. 3).

Fig. 3a displays the long-term polyS accumulation in each reactor. In R1, polyS was found to accumulate throughout the 100 days of operation, from 76.0 mg S/g VSS on Day 1 to 620.0 mg S/g VSS on Day 100, indicating that a higher C/S^0^ ratio (i.e., 0.28) induced more polyS accumulation in sludge. The polyS accumulations in R2 and R3 primarily increased from 77.8 to 71.5 mg S/g VSS on Day 1 to 544.6 and 483.7 mg S/g VSS on Day 80 during phase I and then decreased to 441.7 and 350.6 mg S/g VSS on Day 100 during phase II. For R4, the polyS accumulation remained at low values between 80 and 159 mg S/g VSS during the first 100 days of operation, suggesting that the influent C/S^0^ ratio of naught promoted little polyS accumulation. By combining the changes in sulfate, S^0^ and sulfide metabolism mentioned in Section 3.1.2, it was speculated that under a high influent C/S^0^ ratio (e.g., 0.28 in R1), the sulfur oxidation was more likely to form polyS rather than sulfate [14,27], which correlated well with the observation that polyS accumulated and anoxic sulfate production decreased. Under low influent C/S^0^ ratios (e.g., 0.07 in R3 and naught in R4), fewer carbon sources were retained to induce anaerobic sulfate/S^0^ reduction by the SRB/S^0^RB to generate polyS. This enabled the SOB to efficiently use S^0^ as an electron donor to form sulfate followed by the gradual decrease in the polyS accumulation in R3 and R4.

The glycogen accumulation in R2 showed an upwards trend during phase I (see Fig. 3b), increasing from 3.4 mmol C/g VSS to 4.0 mmol C/g VSS, while the values in the other reactors (i.e., R1, R3, and R4) decreased from 3.7, 3.7, and 4.2 mmol C/g VSS to 1.3, 2.7, and 2.6 mmol C/g VSS, respectively. During phase II, the glycogen accumulations in R1–R4 remained relatively stable at 1.1, 3.7, 2.8, and 2.5 mmol C/g VSS on average. Previous studies have reported that the glycogen content in the biomass can reflect the growth status of GAOs and that a higher glycogen accumulation represents a higher proportion of GAOs in GAO-containing systems [28]. In the present study, the glycogen content of R2 showed an overall upwards trend and was the highest among the four reactors during phase II, which possibly pointed to a higher amount of GAOs in this reactor compared with other reactors. Besides, the glycogen contents in R1, R3 and R4 displayed decreasing trends, suggesting that the proportions of GAOs also decreased. These phenomena again showed that when the influent C/S^0^ ratio was 0.28 in R1, S^0^ weakened the competitive advantage of GAOs; when the influent C/S^0^ ratio was low (e.g., 0.07 in R3 and 0 in R4), the insufficient carbon sources in the influent strongly affected the metabolism of all heterotrophic microorganisms, including GAOs.

The PHA accumulation in R1–R4 mostly showed a decreasing trend throughout the long-term operation (see Fig. 3c). For instance, the PHA amount decreased from approximately 1.0 mmol C/g VSS in all four reactors on Day 1 to 0.27 (R1), 0.55 (R2), 0.28 (R3), and naught (R4) mmol C/g VSS on Day 100 throughout the entire operating period. In addition, the anaerobic production of PHA in R1 with higher sulfide production was lower than that in R2, although R1 had a higher influent C/S^0^ ratio. This decrease was ascribed to the fact that a part of the carbon sources was used for polyS production and glycogen replenishment rather than PHA synthesis [10]. As reported, PHA was used by functional bacteria as the energy and electron donor for N and P removals under the subsequent anoxic phase [26,29], while R2, with its higher PHA production, did not exhibit higher P removal; this indicated that the PHA produced in R2 was mainly used by GAOs. At the same time, higher PHA accumulation was usually accompanied by high glycogen levels (e.g., in R2), which was also used by the GAOs for their growth [13].

### 3.2 Effects of influent C/S^0^ ratios on cyclic performance

The cyclic tests in R1–R4 on Day 92 were selected to display the cyclic profiles with different C/S^0^ mass ratios (see Fig. 4). The biological conversions in terms of C, N, P, and S metabolism and the formation of internal polymers clearly displayed the hybrid SMB and GAO phenotypes in R1–R4. Specifically, it was found that the S metabolism in terms of anaerobic sulfate reduction and S^2−^ production in R1 was significantly different from that in other reactors, which was also supported by the changes in pH and ORP (see Fig. S5 in SI). In R1, nearly no sulfate reduction was found during the anaerobic stage, but 174.7 mg S/L S^2−^ was produced, which included a polyS/S^0^ decrease of 34.9 mg S/g VSS. During the subsequent anoxic stage, S^2−^ was rapidly oxidized to form polyS/S^0^ and sulfate. The metabolism of polyS/S^0^ in R1 was significantly different from a typical DS-EBPR system in which polyS/S^0^ increases during the anaerobic phase and decreases during the anoxic phase. In comparison, the reactions of sulfate or S^0^ reduction in R2–R4 nearly disappeared during the anaerobic stage, and 40.5, 52.4, and 68.2 mg S/g VSS of polyS/S^0^ was consumed, respectively, during the anoxic stage, which was accompanied by 50.3, 68.3, and 106.7 mg S/L sulfate production. These results indicated that the high influent C/S^0^ ratio of 0.28 in R1 supported the growth of both the S^0^RB and SOB, while the relatively low influent C/S^0^ ratios of 0.14, 0.07, and naught in R2, R3, and R4, respectively, limited the growth of S^0^RB or SRB given that the influent carbon sources were insufficient (≤ 200 mg COD/L). The abundance and activity of SOB were maintained since 0.53 g S^0^ was added for the AD reaction in each cycle. In addition, as shown in Table 2, the ratios of Gly/HAc and PHA/HAc in R1 were the lowest, indicating that the carbon source was mainly used for the reduction of S^0^ under high influent C/S° conditions rather than for the synthesis and metabolism of intracellular carbon sources. Meanwhile, R2 and R3 had higher Gly/HAc and PHV/PHA than R1 and R4, which implied that under a low influent C/S^0^ ratio, the GAOs had higher activities than SMB. As reported, the proliferation of GAOs is accompanied by significant PHV production and glycogen hydrolysis during the anaerobic phase [28,30].

**Table 2:** **Stoichiometric ratios and kinetic rates in typical SBR-cycles**.

	Stoichiometric ratios				Kinetic rates*					References
	P_rel_/HA_upt_ (mg P/mg C)	PHA_form_/HAc_upt_ (mg C/mg C)	Gly_form_/HAc_upt_ (mg C/mg C)	PHV_form_/PHA_form_ (mg C/mg C)	HAc_upt_ (mg C/g VSS/h)	PHA_form_ (mg C/g VSS/h)	PHA_deg_ (mg C/g VSS/h)	NO_3con_ (mg N/g VSS/h)	S_oxid_ (mg S/g VSS/h)	
R1	0.017	0.15	0.27	0.00	2.41	0.74	2.56	5.06	0.45	This study
R2	0.004	0.79	0.83	0.14	6.21	3.13	8.24	11.52	9.53	
R3	0.077	1.42	1.48	0.34	1.98	2.78	4.56	3.72	3.67	
R4⁎⁎	–	–	–	–	–	–	–	0.33	0.59	
S-EBPR	0.62	0.58	0.60	0	–	–	–	–	–	[27]
PAO	1.29	1.33	0.50	0	–	–	–	–	–	[31]
GAO	0	1.86	1.12	0.25	–	–	–	–	–	[32]
DPAO	0.41	1.37	0.50	0.20	–	–	–	–	–	[11]
DGAO	0	1.87	1.15	0.25	–	–	–	–	–	[33]

⁎ The kinetics rates were calculated from the first hour of the obtained data in typical SBR-cycles to represent the maximum kinetics rates.

⁎⁎ No acetate was added to R4, so the relevant data are not available.

Regarding the reaction kinetic rates, as shown in Table 2, it was observed that the maximum anaerobic HAc uptake rates in the typical cyclic tests of R1–R4 were 2.41, 6.21, and 1.98 mg C/(g VSS·h) in R1, R2, and R3, respectively, and naught in R4 and that the PHA production and degradation rates in R2 were also higher than those of the other reactors, indicating that the biomass in R2 had much higher acetate uptake and conversion rates than those of R1, R3, and R4. As previously reported [9], when SMB and GAOs were added together in one system, they displayed different abilities to compete for carbon, and SMB had a lower carbon uptake rate than GAOs. Therefore, it can be speculated that R1, R3, and R4 had lower GAO activities than R2. The high influent C/S^0^ ratio of 0.28 in R1 promoted the proliferation of SMB and reduced the activities of GAOs, and the low influent C/S^0^ ratio of 0.07 in R3 and naught in R4 inhibited the activities of all heterotrophic microorganisms, including GAOs and SMB [34,35]. In addition, the anoxic nitrate consumption rate and sulfur oxidation rate of R2 were 11.52 mg N/(g VSS·h) and 9.53 mg S/(g VSS·h), respectively, which were significantly higher than those of the other reactors. This indicated that the influent C/S^0^ ratio of approximately 0.14 in R2 promoted N removal by mixotrophic denitrification, including the S^0^-based autotrophic denitrification performed by SOB coupled with heterotrophic denitrification performed by GAOs.

All the above results correlated well with the long-term performance of R1–R4. R1 displayed higher S^0^RB and SOB phenotypes than R2, R3 and R4; R3 and R4 showed a SOB phenotype over GAOs; and R2 displayed a GAO phenotype over SMB.

### 3.3 Influent C/S^0^ ratios shaped the microbial community structure

To monitor the long-term effects of the influent C/S^0^ mass ratios on the changes in the microbial community structure in R1–R4, fifteen sludge samples were taken on Days 1, 10, 50, 80 and 100 and analysed by 16S rRNA high-throughput sequencing (see Fig. 6). After filtering and trimming of adapters, the barcodes and primers for quality, the effective sequence number, ASV number, and Shannon, Simpson, and GOOD coverage of each sample were obtained and are shown in Table S2. The coverage index in each sample was more than 0.98, indicating that the sequencing results well represented the authenticity of the microorganisms in the samples [36]. The Shannon index of R1 increased from Day 1 to Day 80 during phase I and decreased from Day 80 to Day 100 during phase II, indicating that the microbial diversity of R1 increased first and then decreased [37]. The Shannon index in R2–R4 showed increasing trends throughout the experiments, indicating that relatively low C/S^0^ ratios (≤ 0.14) improved the microbial diversity of these hybrid systems.

Besides, given that the fundamental components of the biological process are the microorganisms involved and their abundance changes in the presence of external environmental factors and operating conditions, in this study, microbial differences were analysed using principal coordinates analysis (PCoA) (see Fig. S6 in SI). Each point on the PCoA map represents a different sample, and the closer the distance between any two points, the lower the microbial difference between them [38]. Based on this interpretation, the microbial communities of the samples taken from R1–R4 on Day 1 and Day 10 had significant similarities. Notably, on Day 50, the distance increased significantly, indicating that the microbial community structures became different. Up to Day 100, the microbial communities in R1–R4 consisted of three groups of distinct clusters, suggesting that the microbial community structures became stable.

The shift in the microbial community structure at the phylum level was measured in the seed sludge and in R1–R4, as shown in Fig. S7. The seed sludge was enriched with 44.7% of *Proteobacteria*, 23.8% of *Bacteroidetes* and 16.4% of *Chloroflexi*, respectively. After 100 days of operation, the relative abundances of *Proteobacteria* in R1–R4 all increased and reached high values of 63.6% (R1), 94.0% (R2), 95.9% (R3) and 84.2% (R4), indicating that *Proteobacteria* became the most dominant microbial community. At the same time, the relative abundances of *Bacteroidetes* and *Chloroflexi* in R1–R4 decreased to nearly undetectable values. Specifically, *Epsilonbacteraeota* was found in R1 on Day 100, and the bacterium *Acidobacteria* appeared in R4 on Day 100. Previous studies have reported that *Epsilonbacteraeota* were able to carry out carbon and nitrogen fixation, nitrate and nitrite reduction and polysulfide reduction [39], and *Acidobacteria* generally appeared in a low carbon environment [40].

**Fig. 5:**
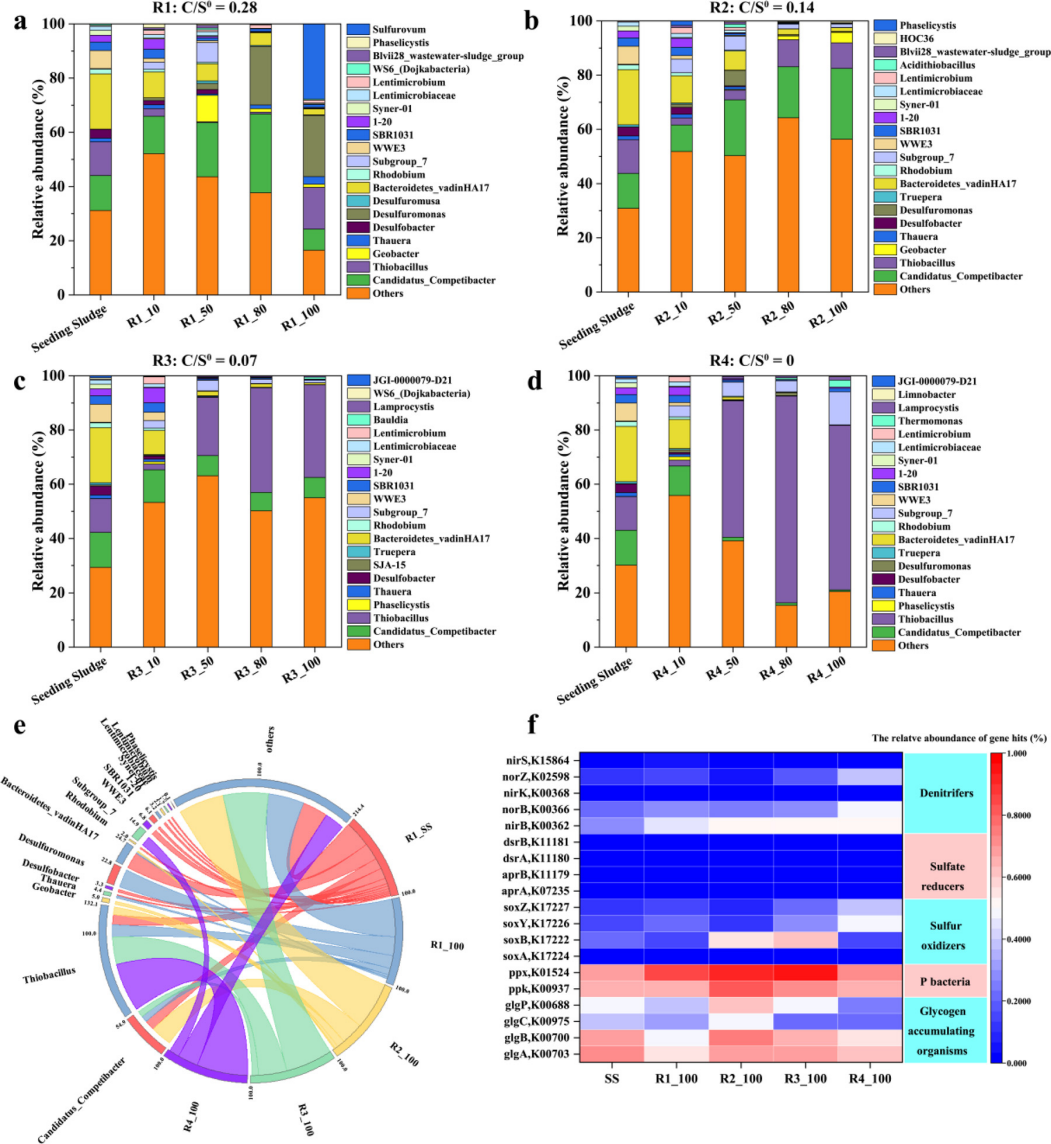
Analysis of microbial community at genus level in reactor (a) R1, (b) R2, (c) R3, (d) R4; (e) microbial community at genus level on day 100; (f) the relative expressions of encoding genes for functional enzymes on day 100.

The shift in the microbial community structure at the genus level was also monitored in R1–R4 to gain an in-depth understanding on the microbial dynamics under different operational conditions, which is presented in Fig. 5. The microbial communities of the seed sludge taken from the parent SBR were found to be enriched by 20.3% with *Bacteroidetes_vadinHA17*, 12.9% with *Candidatus_Competibacter*, 12.5% with *Thiobacillus*, 6.6% with *WWE3*, and 3.2% with *Desulfobacter*. Among them, *Candidatus Competibacter* are typical GAOs, and *Thiobacillus* and *Desulfobacter* are typical SOB and SRB, respectively [9,34]. During the long-term operation, the microbial communities of R1–R4 significantly changed with the different influent C/S^0^ mass ratios. In R1, the relative abundance of the GAO *Candidatus_Competibacter* decreased from an initial 12.9% to 7.8% on Day 100, while the SOB *Thiobacillus* increased from 12.5% to 15.4%; the dominant SRB was transformed from *Desulfobacter* to *Desulfuromonas,* a group of S^0^RB with the ability to reduce S^0^ to S^2−^, which accounted for 22.6% of the total bacteria, indicating that S^0^RB grew well (see Fig. 5a). In R2, the abundances of *Desulfobacter* and *Thiobacillus* decreased to 0.2% and 9.5%, respectively, while *Candidatus Competibacter* increased to 26.1%, indicating that GAOs became the dominant bacteria (see Fig. 5b). For R3 and R4, the relative abundances of the SRB *Desulfobacter* decreased to undetectable levels, and the GAO *Candidatus_Competibacter* decreased to 7.6% in R3 and to 0.5% in R4; the SOB *Thiobacillus* increased and became the dominant bacteria, with the abundances of 34.2% in R3 and 60.7% in R4 (see Fig. 5c and d). The reduction in GAOs was due to the potential adverse effect of sulfide at concentrations higher than 200 mg S/L in R1, and a carbon source deficiency existed in R3–4, inhibiting their growth (see Fig. 2) [10,24]; on the other hand, SOB and SRB were able to utilize available sulfide/S^0^ as substrates for metabolism [22]. As a result, under a high influent C/S^0^ ratio (i.e., 0.28 in R1), sulfide/S^0^ became an additional electron donor/acceptor for SMB (i.e., SRB/S^0^RB or SOB), increasing their relative abundance and activity [4,5]. When the influent carbon source was insufficient (i.e., C/S^0^ ratio of 0.07 in R3 and naught in R4), the relative abundances of the heterotrophic microorganisms (including SRB/S^0^RB and GAOs) decreased, while only autotrophic SOB were dominant in the presence of sufficient S^0^. In contrast, when the influent C/S^0^ ratio was 0.14, with 200 mg COD/L and 0.53 g S^0^ addition, GAOs were favoured over SMB as they competed for limited carbon sources, and they became dominant, particularly in R2.

The key genes involved in various metabolic pathways, including dissimilatory sulfate reduction and sulfide/sulfur oxidation (*sox, aprAB* and *dsrAB*), denitrification (*nirS, norB* and *nosZ*), glycogen conversion (*glyABC* and *glyP*), polyphosphate conversion *ppx* (exopolyphosphatase), and *ppk* (polyphosphate kinase), in the seed sludge and samples from R1–R4 on Day 100 were analysed, as shown in Fig. 5f. The abundance of a particular gene reflects the capacity of a particular microbe to metabolize C, P, N, and S. The results showed that the relative abundances of the related genes representing SRB/S^0^RB, SOB, GAOs, denitrifiers, and “PAOs” changed with the influent C/S^0^ ratios [24]. For instance, the relative abundances of the SRB genes (including *aprAB* and *dsrAB*) in R1–R4 were lower than 0.1 ‰, those of the SOB genes (including *sox*) were 0.57 ‰ (R1), 0.76 ‰ (R2), 1.10 ‰ (R3), and 1.07 ‰ (R4), while those of the GAOs genes (including *glyABC* and *glyP*) were 1.72 ‰ (R1), 2.54 ‰ (R2), 2.04 ‰ (R3), and 1.65 ‰ (R4). These results correlated well with the changes in the microbial community structures in the four reactors, in which the influent C/S^0^ ratios of 0.14 and 0.07 in R2 and R3, respectively, were accompanied by an increase in S^0^ addition, promoting the abundances of SOB genes; additionally, significantly high or low influent C/S^0^ ratios (i.e., 0.28 in R1 and naught in R4) limited the abundances of the GAO genes.

### 3.4 Influent C/S^0^ ratios favoured SMB over GAOs

**Fig. 6:**
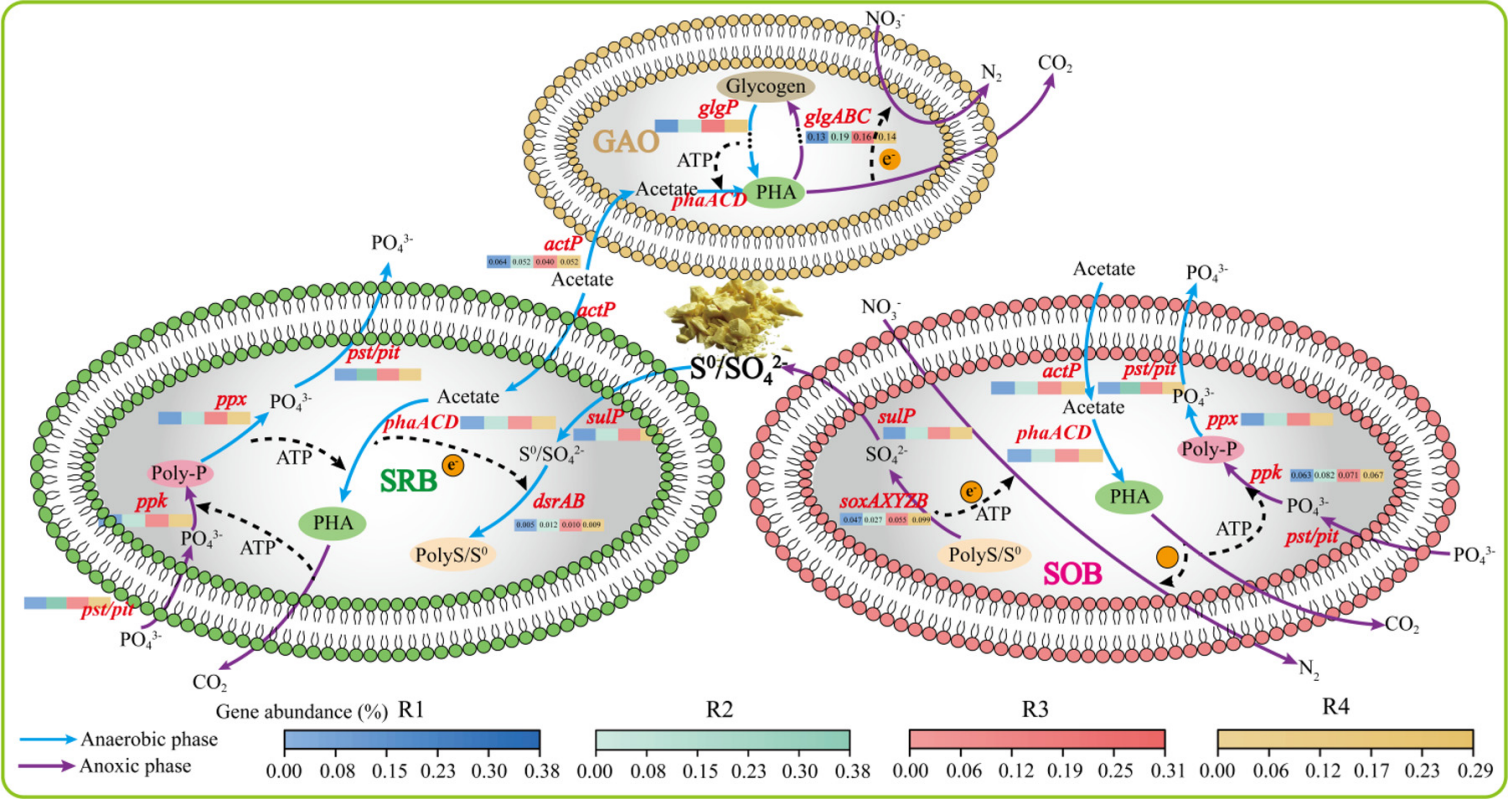
**Schematic illustration of the potential mechanism**.

The potential mechanisms by which the different C/S^0^ mass ratios affected the competition between SMB and GAOs were illustrated (see Fig. 6) in the above results and discussion on C, N, P, and S metabolism, internal polymers, cyclic performance, and shift in microbial community structure. In a single cycle within this hybrid system, under anaerobic conditions, SMB (i.e., SRB/S^0^RB and SOB) and GAOs competed for carbon sources for 1) sulfate or S^0^ reduction, 2) synthesis of internal polymers (e.g., PHA and polyS) accompanied by glycogen degradation, and 3) growth of SMB or GAOs. At the same time, specific types of SMB yielded little P release. Under subsequent anoxic conditions, SMB (especially SOB) can utilize polyS/S^0^ and PHA as energy and electron donors, respectively, and nitrate as the electron acceptor to perform N and P removals; while GAOs can only use PHA as the internal carbon source for glycogen replenishment and N removal without P removal [9,24]. Generally, GAOs had an advantage in competing for carbon sources given that they had a higher carbon uptake rate than SMB [9], which could be the reason for the enrichment in GAOs in R2 when the amount of carbon sources became limiting. The relevant genes encoding key enzymes involved in converting PHA into glycogen could also support the advantage of GAOs. For instance, the total abundance of *glgABC* in R2 was 0.19%, higher than that of 0.13%−0.16% in other reactors (Fig. 6), resulting in an enrichment of GAOs in R2.

In R1–R4, the different influent C/S^0^ mass ratios were achieved by carbon source regulation (i.e., 400, 200, 100, 0 mg COD/L) and the addition of a fixed amount of S^0^ (0.53 g). Once S^0^ was added to the reactors, it was used in S metabolism. In the present study, a sufficient dose of S^0^ was required to promote the growth of SMB, or in some cases, no S^0^ was needed to replace the influent carbon source (e.g., 400 mg COD/L in R1 or naught in R4) to avoid putting the SMB at a disadvantage relative to GAOs during the start-up phase. Specifically, S^0^RB in R1 can use added S^0^ to achieve anaerobic S^0^ reduction to form an intermediate product, i.e., polyS, and subsequently HS^−^/S^2−^ [41,42]. Studies have found that polyS is more hydrophilic and more easily utilized by SRB than S^0^ [26]. The HS^−^/S^2−^, generated by S^0^ reduction, promoted the growth of SRB but limited the growth and activity of GAOs [22,42]. Then, under anoxic conditions, polyS/S^0^ or HS^−^/S^2−^ were oxidized to SO_4_^2−^ by SOB, activating and enhancing SOB activity. In R4, given that there was no carbon source in the influent, GAOs and SRB/S^0^RB had no carbon source to use and were quickly eradicated, while SOB (e.g., *Thiobacillus*) used S^0^ as the energy source for their growth. In comparison, in R2 and R3, which had 200 and 100 mg COD/L added to their influent, respectively, the GAOs relied on the limited carbon available for maintenance and growth, even though S^0^ was added; therefore, the GAOs were not eradicated.

Despite the strong evidence presented in this study to support the abovementioned mechanism, the mechanisms of internal polymer conversion, microbial community shifts, and complete metabolic competition between SMB (including SRB/S^0^RB and SOB) and GAOs have yet to be revealed. The main issues that need to be addressed on the role of P and GAOs in the C, N, and S metabolisms are 1) the reasons why the P removal efficiencies in R1 and R4 did not recover relative to a normal DS-EBPR, although these reactors had a higher abundance of SOB; 2) methods to improve the contribution of GAOs to C and N removals without weakening the P removal efficiency driven by SMB; 3) methods to strengthen the role of polyS/S^0^ as energy and electron carriers in the P removal process to better replace carbon polymers (PHA and glycogen); that is, further studies are required to elucidate the characteristics of polyS/S^0^ generation, conversion, and utilization. More investigations are needed to address these issues.

## 4 Implication

Biological nutrient (including N and P) removal is often limited by deficiencies in organic carbon sources, especially in southern China. A DS-EBPR process was developed to use inexpensive sulfur sources (e.g., SO_4_^2−^, S^2−^, S^0^) to partially replace carbon in the simultaneous removal of N- and P-containing pollutants [19]. When operated effectively, this process could be an inexpensive option to achieve relatively high N and P removal while limiting sludge production. However, similar to the conventional EBPR process, the DS-EBPR process can malfunction and lead to the excessive growth of GAOs in some cases. To develop control strategies to suppress the proliferation of GAOs, it is key to understand the mechanisms by which the operating or environmental conditions affect the competition between SMB (i.e., S^0^RB/SRB and SOB) and competing GAOs.

For the first time, the optimal operating conditions needed for SMB to outcompete GAOs were investigated under different influent C/S^0^ ratios and are presented in this study. The results showed that S° can not only act as an electron donor to partially replace carbon in nutrient removal but also improve the competitiveness of SMB over GAOs. S^0^ addition efficiently increased the activities of SMB (especially anaerobic S^0^ reduction and S^0^ oxidation) when the influent C/S^0^ ratios were high (0.28 in R1) or low (0.07 in R3 or naught in R4). Particularly in R1, sulfide was produced by S^0^RB, which were able to inhibit the growth of GAOs and increase the competitiveness of SMB [14,22]. In this work, the nitrogen removal rate was 0.024–0.36 g N/L/d, similar to that reported in previous literature (Table S3, SI), suggesting that S^0^-assisted EBPR is expected to enhance nutrient removal in WWTPs, especially in carbon-limited biological systems. Unfortunately, while the abundance of GAOs and related genes in R1, R3 and R4 were reduced, the P removal performance improved only marginally (Table S3, SI). This is consistent with the previous finding that once sulfur-driven P removal performance deteriorates or fails, it is hard to restore [10]. Besides, considering the limited bioavailability of S^0^ [5], some approaches, such as the use of modified colloidal S^0^, sulfur beads and S^0^-packed biological stuffing, can be adopted to avoid S^0^ sedimentation, accelerate its biotransformation and improve the nutrient removal performance [4]. The strategies used in this study 1) prevented the excessive growth of GAOs and 2) improved N and P removal performances and can offer a novel approach for supporting the growth of SMB over GAOs.

## 5 Conclusion

In this study, the long-term effects of different influent C/S^0^ ratios on the competition for carbon sources between SMB and GAOs in carbon-deficient wastewater were investigated. The main conclusions are as follows:

(1) The substrate S^0^ acted as the additional electron donor to improve the competitiveness of SMB and inhibited the excessive growth of GAOs.
(2) A relatively high influent C/S^0^ ratio of 0.28 mg C/mg S or relatively low values less than 0.07 mg C/mg S enhanced S metabolism performed by SMB, which outcompeted GAOs.
(3) GAOs had an advantage over SMB in competing for limited carbon sources when the influent C/S^0^ ratio was kept at 0.14 mg C/mg S, even in the presence of S^0^.

## Declaration of competing interest

The authors declare that they have no conflicts of interest in this work.

## Appendix Supplementary materials

Image, application 1
