# Supplementary material for: Sulfur-mediated bacteria outperform glycogen-accumulating organisms in carbon-deficient wastewater: Key role of influent C/S0 ratios
Source: Fundam Res. 2024 Jan 14;6(1):212–22. doi: 10.1016/j.fmre.2023.10.024 (PMC12869783; doi:10.1016/j.fmre.2023.10.024)
Supplement: Supplementary file 1 [file mmc1.docx]

**Supporting Information**

# Sulfur-mediated bacteria outperform glycogen-accumulating organisms in carbon-deficient wastewater: key role of influent C/S^0^ ratios

Boyi Cheng^a,1^, Lei Chen^a,1^, Lichang Zhou^a^, Qingshan Lin^a^, Jinqi Jiang^a^, Hui Lu^b,^*, Lei Miao^a^, Xiaonan Feng^a^, Zongping Wang^a^, Guanghao Chen^c^, Gang Guo^a,^*

^a^ School of Environmental Science and Engineering, Huazhong University of Science and Technology (HUST), Wuhan, 430074, China

^b^ School of Environmental Science and Engineering, Sun Yat-sen University, Guangzhou, 510006, China

^c^ Department of Civil & Environmental Engineering, The Hong Kong University of Science and Technology, Hong Kong, China

*Corresponding author: Dr. Hui Lu, E-mail: [lvhui3@mail.sysu.edu.cn](mailto:lvhui3@mail.sysu.edu.cn); Dr. Gang Guo, e-mail: [ceguogang@hust.edu.cn](mailto:ceguogang@hust.edu.cn)

^1^ Contributed equally to the work


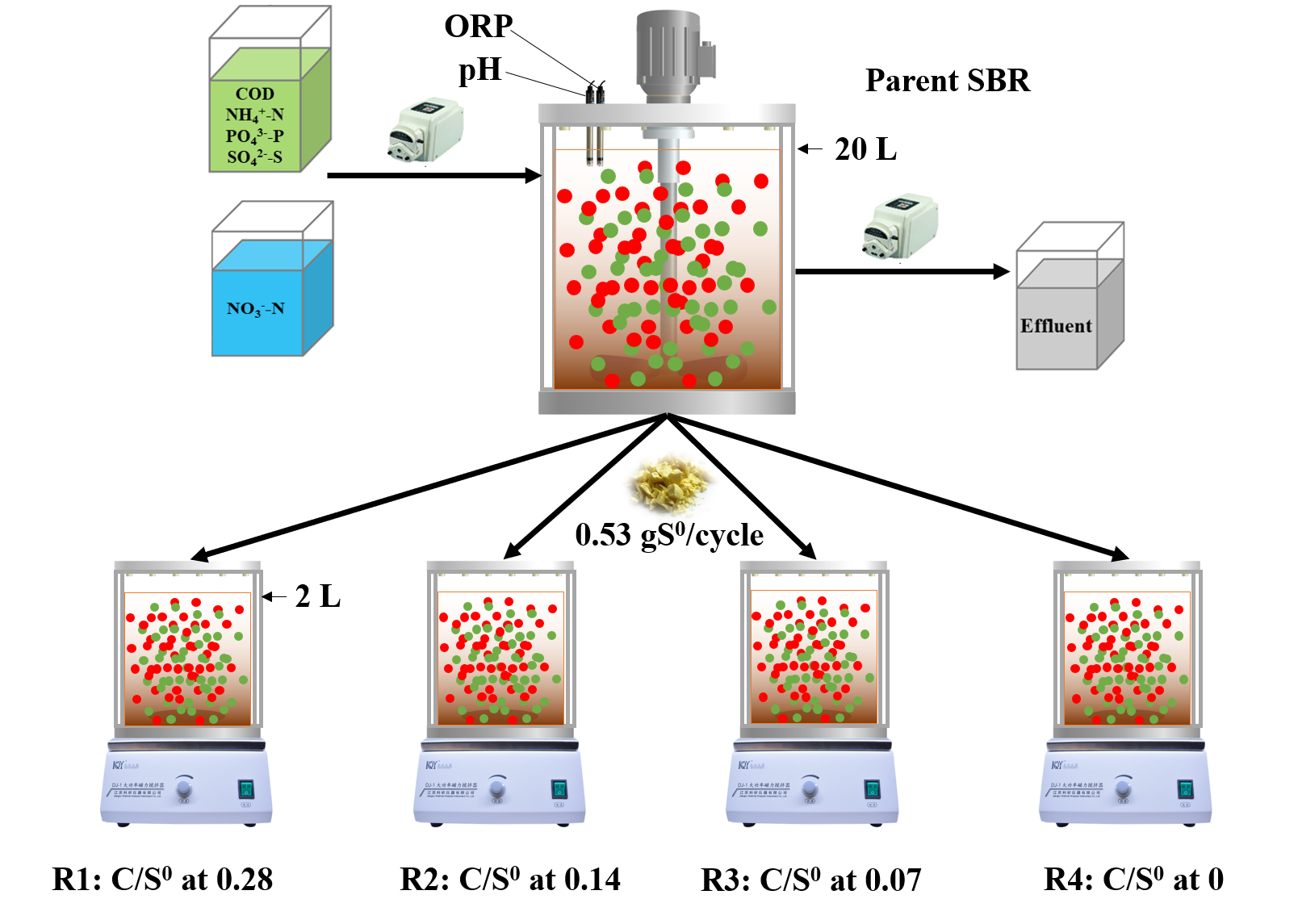


Fig. S1 Schematic diagram of experimental reactors.

**Supplementary Information 1: XRD analysis of the sludge samples**

In this experiment, the sludge samples were collected from the enrichment experimental reactor (D0), without elemental sulfur addition, and from the batch reactors (R1–R4), on Day 100, with elemental sulfur addition (i.e. R1_100, R2_100, R3_100, and R4_100). The XRD analysis is shown in Fig. S2. The XRD pattern of sample D0 had a strong diffraction peak, which was consistent with the standard SiO_2_ diffraction peak (PDF#79-1906). Besides, the XRD patterns of samples R1_100, R2_100, R3_100, and R4_100 had a series of peaks, which were basically consistent with the S_8_ standard spectrum (PDF#74-1465). The peak intensity of S_8_ in R1–R4 clearly changed, indicating that the sulfur accumulation in the sludge samples had decreased, which was consistent with the variation in the polysulfide accumulation. The XRD analysis further showed that the changes in the influent carbon source content significantly affected the utilization efficiency of elemental sulfur. When the influent carbon source content was 400 mg COD/L, elemental sulfur was consumed during the anaerobic stage and generated during the anoxic stage. The amount of dosage of elemental sulfur was always higher than the consumption of it, resulting in the accumulation of elemental sulfur in the sludge samples. Meanwhile, the lower the influent carbon source content, the faster the propagation of SOB in the reactor. This led to the continuous conversion of elemental sulfur into sulfate, which in turn resulted in the drop in the sulfur accumulation.


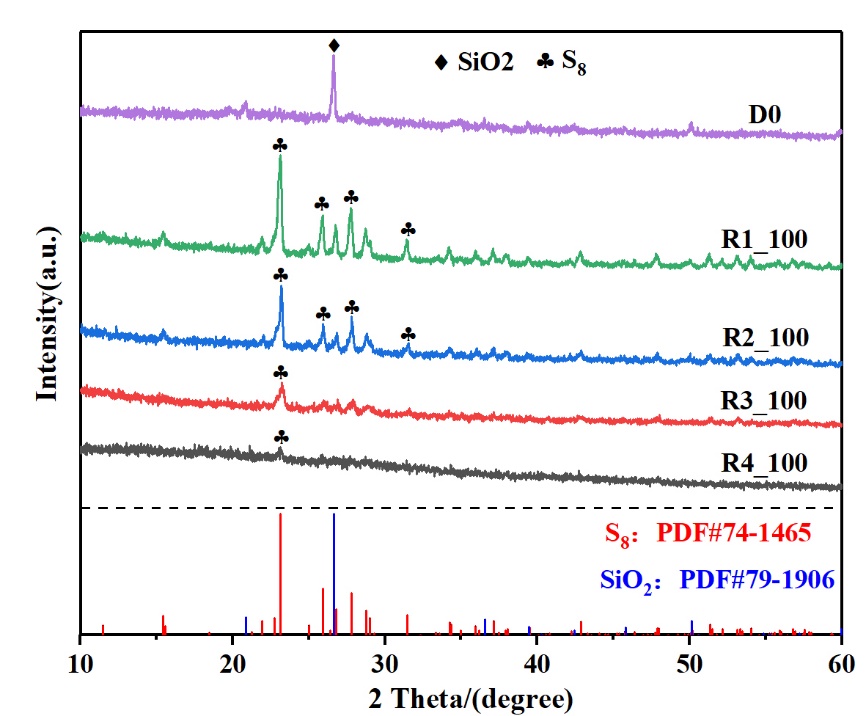


Fig. S2 XRD analysis of sludge samples.

### Supplementary Information 2: XPS analysis of the sludge samples

Fig. S3 shows that the main elements in the sludge samples are C, O, N, P, and S, which is consistent with the main components of the sludge samples taken by Guo et al. in the DS-EBPR system [1]. Among those, C, O, and N can represent various kinds of organic and inorganic compounds existing in sludge samples; P can originate from phosphate precipitates; and S can originate from sulfur-containing substances in different combination states. According to the analysis by Zhao et al. [2], narrow spectrum scanning data of sulfur in individual samples were fitted (Fig. S4). The S^2-^content in the sludge sample of R1 significantly increased compared with that before separation. This can be due to the fact that when the influent carbon source concentration was 400 mg COD/L, elemental sulfur promoted the growth of SRB in the long-term experiment. Sulfide was produced in each cycle, which further precipitated with metal elements in the sludge, resulting in an increase in the proportion of S^2-^ form in the sludge samples. When the influent carbon content was 200 mg COD/L, 100 mg COD/L, or when there was no carbon as in R4, the SRB were not able to produce sufficient metal sulfide. On the contrary, elemental sulfur increased the SOB activity in the long-term experiment. Afterwards, the metal sulfide precipitates in the sludge samples were gradually oxidized, resulting in the decrease in S^2-^ content in the samples.


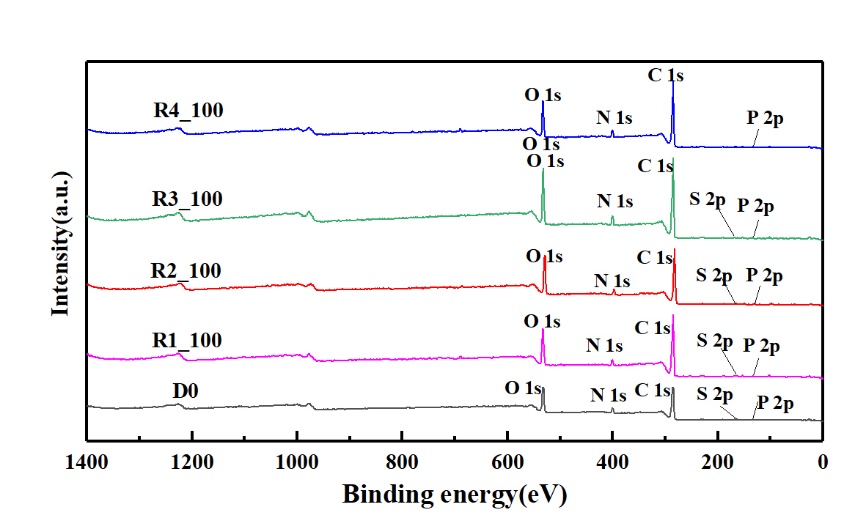


Fig. S3 Full scan XPS spectra of sludge samples.

| 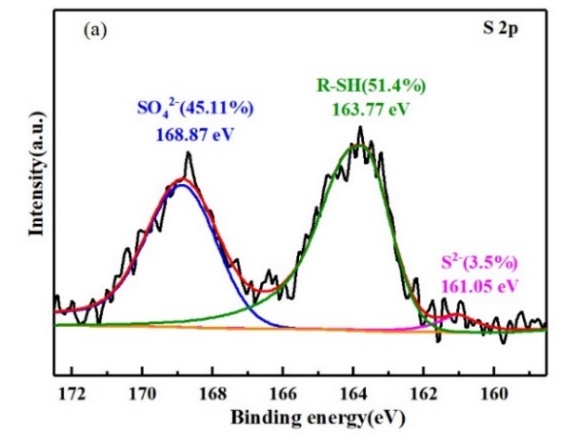 | |
| --- | --- |
| 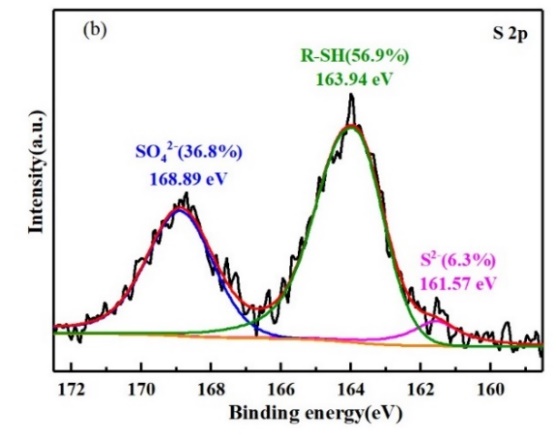 | 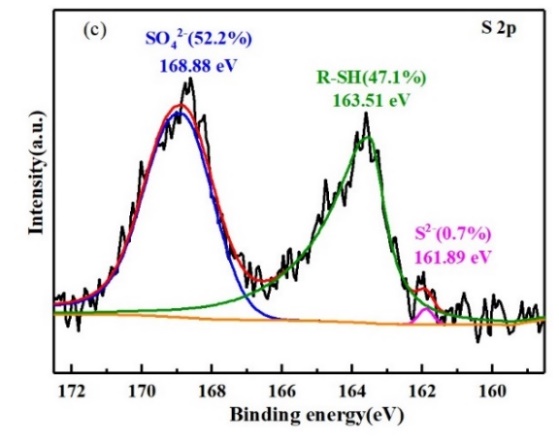 |
| 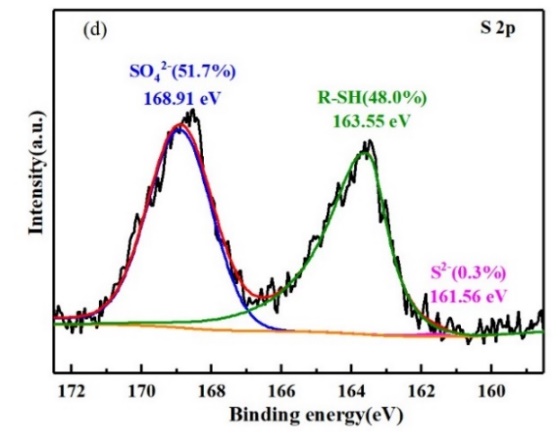 | 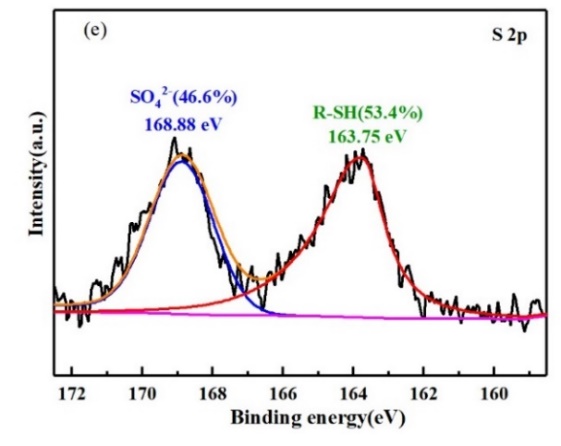 |

Fig. S4 Narrow XPS scanning spectra of sulfur in sludge samples (a) D0, (b) R1_100, (c) R2_100, (d) R3_100, and (d) R4_100.

**Supplementary Information 3: pH and ORP monitoring in cyclic tests**

This study monitored the changes in pH and ORP in typical cycles, as shown in Fig. S5. It showed that the pH of R1 decreased from 7.4 to 6.4 during the anaerobic phase and increased rapidly during the anoxic phase, which was significantly different from that of other reactors. The elemental sulfur during the anaerobic phase of R1 was reduced to sulfide, accompanied by H^+^ formation. Then, the sulfide was oxidized and produced alkalinity during the anoxic phase. As for R2–R4, the sulfide disappeared in the typical cycle. Hence, the pH of R2 and R3 decreased significantly during the anoxic phase. Moreover, research has found that with the decrease in the influent carbon source in each reactor, the amount of reducing substances also decreased [1]. This made the reactor ORP change the less obvious.


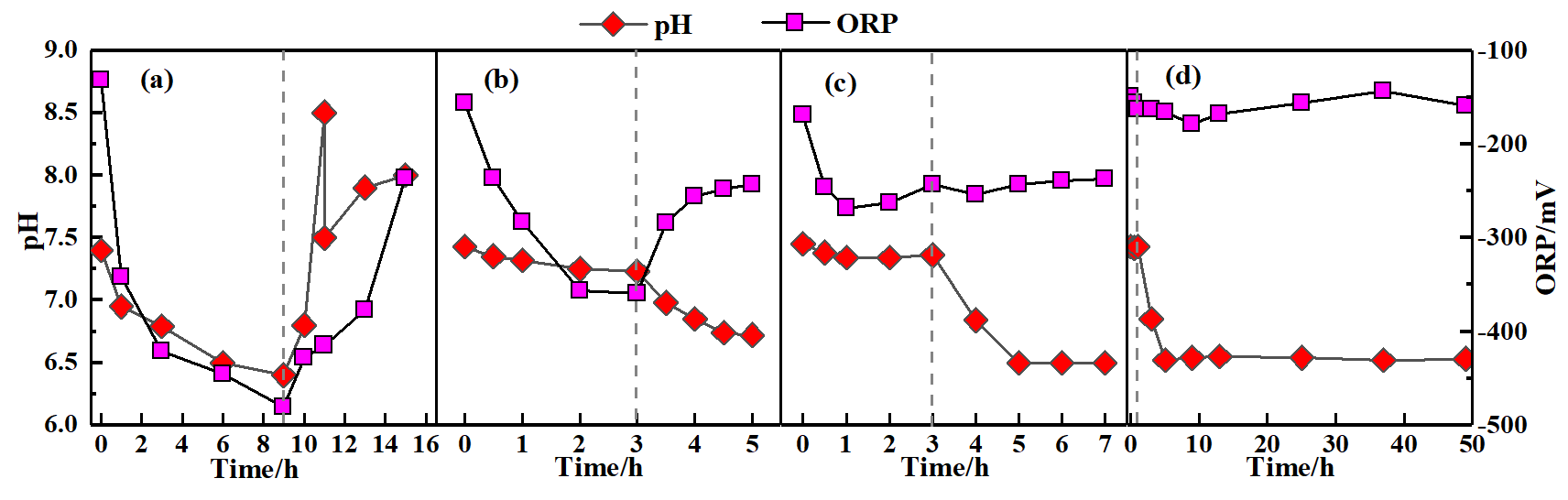


Fig. S5 Changes in pH and ORP in reactors (a) R1, (b) R2, (c) R3, and (d) R4, during typical cycles.


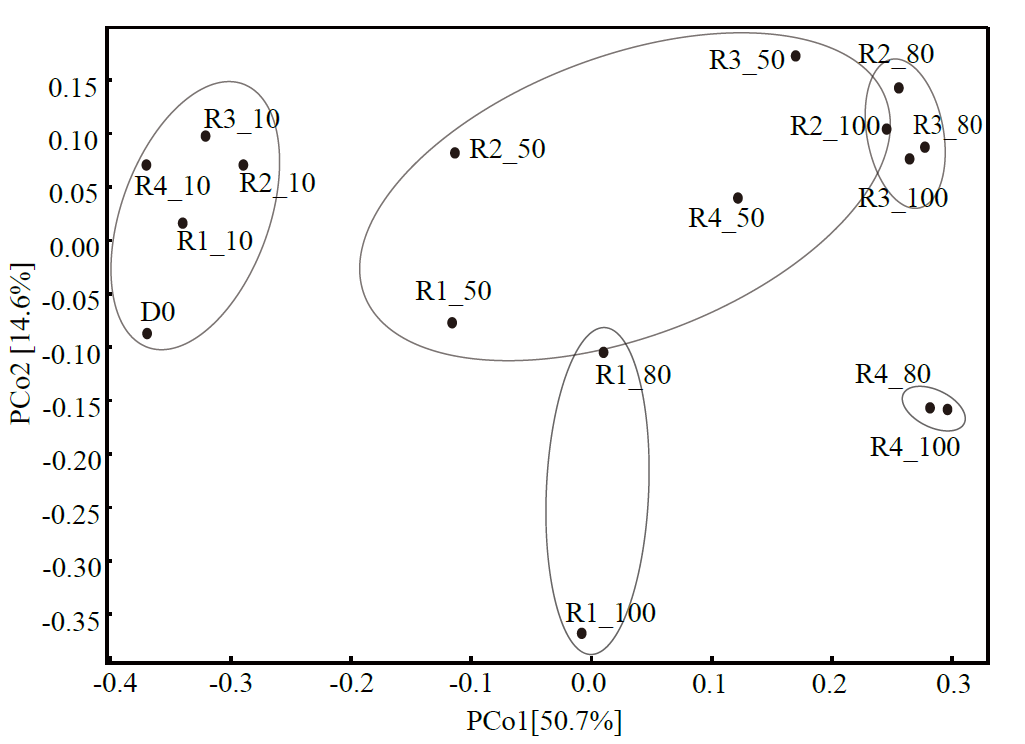


Fig. S6 PcoA analyses of microbial community structures in R1–R4.


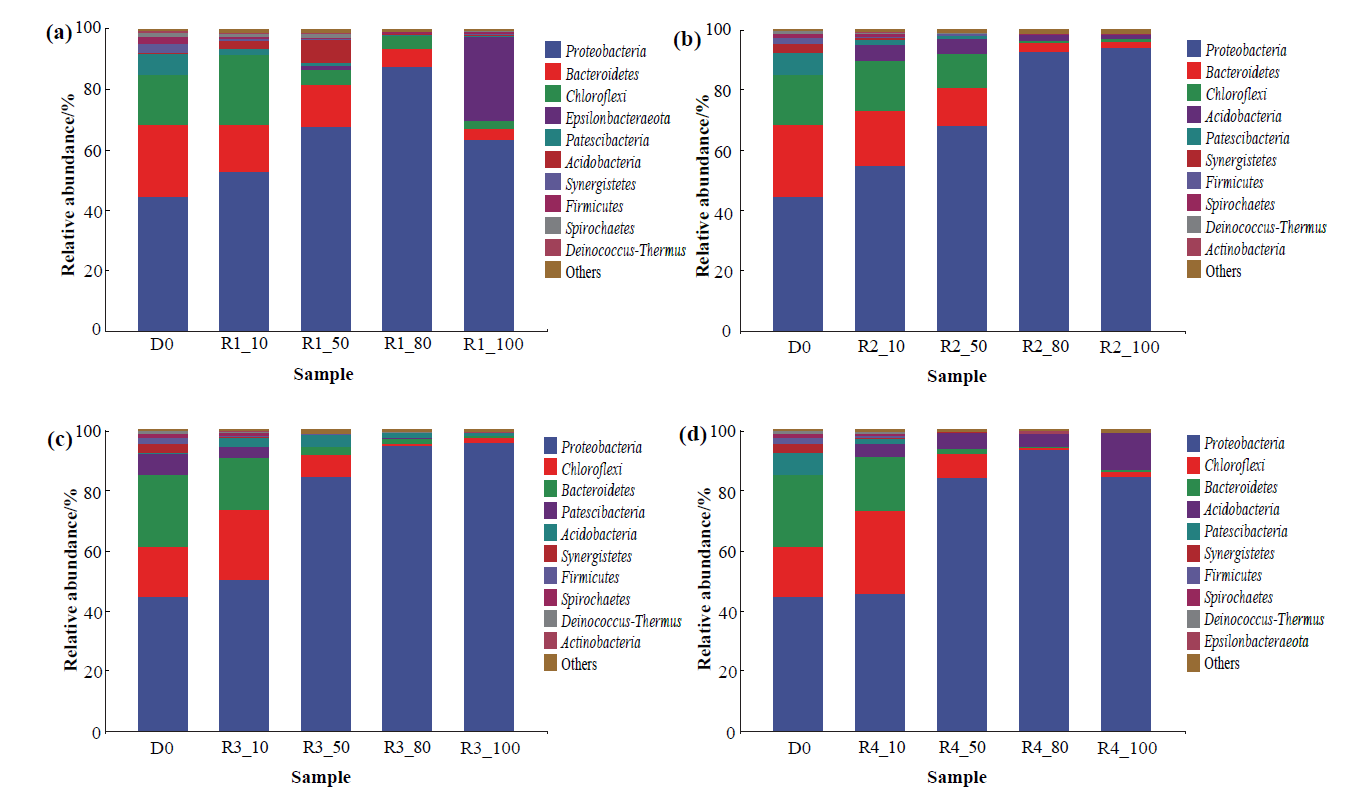


Fig. S7 Analyses of microbial communities at phylum level in R1 (a), R2 (b), R3 (C), and R4 (D).

Table S1 The conditions for the analytical characterization of the sludge.

| Methods | Conditions |
| --- | --- |
| XRD | CuKα radiation; Scan: 10°-80°; Scan speed: 5° 2θ/min; Step width: 0.02° |
| XPS | Binding energy range: 0-1350 eV; Elements: C, O, N, P, S |

Table S2 Comparisons of microbial community abundances and diversities in R1–R4.

| Item | Effective sequence | ASV | Shannon | Simpson | Coverage |
| --- | --- | --- | --- | --- | --- |
| Seeding sludge | 58642 | 317 | 5.0292 | 0.933911 | 0.999503 |
| R1_10 | 63176 | 329 | 5.14737 | 0.935034 | 0.99921 |
| R1_50 | 132492 | 1027 | 5.98022 | 0.947204 | 0.992589 |
| R1_80 | 77324 | 2168 | 7.18125 | 0.949577 | 0.994258 |
| R1_100 | 116455 | 1360 | 5.61092 | 0.901183 | 0.990107 |
| R2_10 | 61495 | 302 | 5.05703 | 0.926357 | 0.999375 |
| R2_50 | 132244 | 1302 | 5.96309 | 0.929231 | 0.989913 |
| R2_80 | 100342 | 2119 | 6.28422 | 0.860002 | 0.985507 |
| R2_100 | 92759 | 2144 | 6.87076 | 0.915646 | 0.987676 |
| R3_10 | 60852 | 358 | 5.10769 | 0.927741 | 0.999176 |
| R3_50 | 127363 | 1296 | 5.11667 | 0.831737 | 0.990035 |
| R3_80 | 128742 | 1027 | 4.74498 | 0.834122 | 0.992646 |
| R3_100 | 88454 | 1752 | 6.58234 | 0.916878 | 0.991515 |
| R4_10 | 57847 | 313 | 4.9305 | 0.921734 | 0.999419 |
| R4_50 | 123461 | 873 | 4.46798 | 0.809315 | 0.993852 |
| R4_80 | 111405 | 1387 | 4.99686 | 0.763491 | 0.988954 |
| R4_100 | 71305 | 1377 | 6.01288 | 0.90215 | 0.994724 |

Table S3 The nutrient removal performance in sulfur-assisted biological nutrient removal systems.

| Reactor type | Wastewater type | Sulfur source | N removal rate  (g/L/d) | P removal rate  (mg P/L/d) | Reference |
| --- | --- | --- | --- | --- | --- |
| SBR | Synthetic wastewater | Elemental sulfur | 0.024-0.36 | 3.84-12 | This study |
| UASB | Industrial wastewater | Thiosulfate | 0.33 | - | [3] |
| Packed bed reactors | Mariculture wastewater | Elemental sulfur | 0.054-0.546 | - | [4] |
| Packed bed reactors | Synthetic wastewater | Elemental sulfur | 1.46-1.65 | - | [5] |
| SBR | Synthetic wastewater | Sulfate | 0.22 | 60 | [6] |
| MBBR | Synthetic wastewater | Sulfide | 0.03 | - | [7] |

**Reference:**

[1] G. Guo, D. Wu, T. Hao, H.R. Mackey, L. Wei, H. Wang, G.H. Chen, Functional bacteria and process metabolism of the Denitrifying Sulfur conversion-associated Enhanced Biological Phosphorus Removal (DS-EBPR) system: An investigation by operating the system from deterioration to restoration, Water Res. 95 (2016) 289-299. https://doi.org/10.1016/j.watres.2016.03.013.

[2] Q. Zhao, J. Wang, S. OuYang, L. Chen, M. Liu, Y. Li, F. Jiang, The exacerbation of mercury methylation by Geobacter sulfurreducens PCA in a freshwater algae-bacteria symbiotic system throughout the lifetime of algae, J. Hazard. Mater. 415 (2021) 125691. https://doi.org/10.1016/j.jhazmat.2021.125691.

[3] Y.F. Deng, G.A. Ekama, Y.X. Cui, C.J. Tang, M.C.M. van Loosdrecht, G.H. Chen, D. Wu, Coupling of sulfur(thiosulfate)-driven denitratation and anammox process to treat nitrate and ammonium contained wastewater, Water Res. 163 (2019) 114854. https://doi.org/10.1016/j.watres.2019.114854.

[4] L. Wang, J. Liu, Y. Li, Z. Liu, L. Zhang, H. Che, H. Cui, Y. Zhang, Elemental sulfur-driven autotrophic denitrification process for effective removal of nitrate in mariculture wastewater: Performance, kinetics and microbial community, Chemosphere 337 (2023) 139354. https://doi.org/10.1016/j.chemosphere.2023.139354.

[5] Y.Y. Qiu, X. Gong, L. Zhang, S. Zhou, G. Li, F. Jiang, Achieving a novel polysulfide-involved sulfur-based autotrophic denitrificationprocess for high-rate nitrogen removal in elemental sulfur-packed bed reactors, ACS ES&T Eng. (2022) 2, 1504-1513. https://doi.org/10.1021/acsestengg.2c00017.

[6] G. Guo, D. Wu, G.A. Ekama, T. Hao, H.R. Mackey, G.H. Chen, Denitrifying sulfur conversion-associated EBPR: Effects of temperature and carbon source on anaerobic metabolism and performance, Water Res. 141 (2018) 9-18. https://doi.org/10.1016/j.watres.2018.04.028.

[7] Y.X. Cui, G. Guo, B.K. Biswal, G.H. Chen, D. Wu, Investigation on sulfide-oxidizing autotrophic denitrification in moving-bed biofilm reactors: An innovative approach and mechanism for the process start-up, Int. Biodeterior. Biodegrad. 140 (2019) 90-98. https://doi.org/10.1016/j.ibiod.2019.03.016.
